# Establishing the Research Agenda for Increasing the Representation of Women in Engineering and Computing

**DOI:** 10.3389/fpsyg.2017.00598

**Published:** 2017-04-19

**Authors:** Kathleen Buse, Catherine Hill, Kathleen Benson

**Affiliations:** ^1^Department of Design & Innovation, Weatherhead School of Management, Case Western Reserve University, ClevelandOH, USA; ^2^Research Department, American Association of University Women, WashingtonDC, USA

**Keywords:** gender equality, women, engineering, computing

## Abstract

While there is an extensive body of research on gender equity in engineering and computing, there have been few efforts to glean insight from a dialog among experts. To encourage collaboration and to develop a shared vision of the future research agenda, a 2 day workshop of 50 scholars who work on the topic of gender in engineering and computing was held at a rural conference center. The structure of the conference and the location allowed for time to reflect, dialog, and to craft an innovative research agenda aimed at increasing the representation of women in engineering and computing. This paper has been written by the conference organizers and details the ideas and recommendations from the scholars. The result is an innovative, collaborative approach to future research that focuses on identifying effective interventions. The new approach includes the creation of partnerships with stakeholders including businesses, government agencies, non-profits and academic institutions to allow a broader voice in setting research priorities. Researchers recommend incorporating multiple disciplines and methodologies, while expanding the use of data analytics, merging and mining existing databases and creating new datasets. The future research agenda is detailed and includes studies focused on socio-cultural interventions particularly on career choice, within undergraduate and graduate programs, and for women in professional careers. The outcome is a vision for future research that can be shared with researchers, practitioners and other stakeholders that will lead to gender equity in the engineering and computing professions.

## Introduction

In 2015 women comprised 25% of computer scientists and 12% of engineers in the USA. This is at a time when women comprise 47% of the total labor force and 52% of managers and professionals. Women’s representation in other demanding professions belie this underrepresentation in engineering and computing as women comprise 63% of auditors and accountants, 54% of business professionals, 38% of physicians and surgeons, and 35% of lawyers (US Bureau of Labor Statistics, 2015).

The topics of science, technology, engineering, and mathematics (STEM) and “women in STEM” continue to attract attention in the public discourse and in academia. Women’s under-representation in these professions has inspired an influx of research over the past four decades, as summarized in recent reports (Hill et al., 2010; Corbett and Hill, 2015). Meta-analyses (Kanney et al., 2014) have identified trends in research, documenting what is known and what needs to be known about gender equity in engineering and computing. Although the problem is well documented, understanding how to overcome the obstacles to women’s full participation in the technical workforce, particularly empirical research on effective interventions, remains a challenge.

The purpose of this paper is to present the ideas and recommendations from a gathering of researchers who worked to create a research agenda to increase the representation of women in engineering and computing. The conference was designed to encourage collaboration among researchers and to craft an innovative research agenda to accelerate the progress of women in engineering and computing. This paper has been written by the conference organizers using detailed notes and session transcripts. The authors intend for this paper to provide the context and the specifics for a concise, prioritized research agenda to be released by the American Association of University Women (AAUW) in the spring of 2017. Together, the paper and the research agenda provide a path forward for researchers and those who depend upon research to increase the numbers of women who pursue, thrive and excel in engineering and computing professions.

This paper begins by explaining the motivation and logistics behind the research conference followed by a high-level overview of the research and a discussion of effective evidence-based interventions. Next the researchers’ vision for a new collaborative approach to the work is described including: the creation of an oversight group; an intentional effort to impact the depth and breadth of research studies by using multiple disciplines, multiple methods and simultaneously studying multi-levels; leveraging data, merging and mining existing data sources, using data analytics to create knowledge and providing opportunities for researchers to convene on a regular basis. Finally, a description is provided of recommended research studies contextually categorized as societal and cultural influences, undergraduate and graduate programs, and the workplace. The outcome is a path forward for researchers, practitioners and other stakeholders that will lead to gender equity in the engineering and computing professions.

## Background

An extensive literature review (Corbett and Hill, 2015) highlighted recent research that identifies the critical factors underlying the under-representation of women in engineering and computing. These factors include stereotypes and biases embedded in society, in college curriculum, and in the workplace. The agenda for the research conference was organized around these factors.

Leading researchers were selected to attend the conference through an application process. Those attending worked as professors and research scientists in fields that spanned engineering, computer science, education, psychology, sociology, gender studies, management, and public policy. Others worked as administrators in higher education or directors of non-profits. All had some connection with research focused on increasing gender equality in engineering and/or computer science, with many having NSF-funded research.

The conference was structured to include large group sessions and small group discussions. Informal gatherings and meals allowed for continued discussion and debate. The large group sessions reviewed logistics, provided overarching goals for the smaller sessions, and the opportunity for the facilitators of the small group to share and synthesize outcomes. The small group discussions were used to create a discourse among the researchers on what we know and what we need to know in three critical areas: (1) the impact of implicit gender bias and stereotype threat on individuals in the context of career choice, persistence in undergraduate and graduate programs, and in the workplace; (2) evidence-based interventions to increase women’s representation in engineering and computing; and (3) creating a specific research agenda. All discussions focused on how research informed, and should inform, practice by employers, policy maker, and individuals.

Included in the body of knowledge of the factors impacting women’s under-representation in engineering and computing are stereotypes, a cognitive shortcut that categorizes people on the basis of characteristics such as gender, race, or age. A bias is a semi-permanent belief based on repeated exposure to stereotypes (Banaji and Greenwald, 2013). A meta-analysis of gender and leader stereotypes found no evidence of decreased stereotyping over time (Koenig et al., 2011).

Both men and women show biases toward women in scientific roles (National Research Council, 2007; Hill et al., 2010; Moss-Racusin et al., 2012; Corbett and Hill, 2015). While overt bias is less common today than in the past, implicit biases remain common (Banaji and Greenwald, 2013). Implicit, or unconscious, bias occurs when a person consciously rejects stereotypes but unconsciously makes evaluations based on stereotypes. The social psychologists Mahzarin Banaji and Anthony Greenwald introduced the concept of implicit bias in 1995, building on earlier findings showing that individuals’ actions are not always under their conscious control. Banaji and Greenwald (2013) believe that implicit bias often expresses itself through in-group favoritism, which can be hard to detect. For example, despite finding no evidence of an explicit preference for male or female managers, researchers found that male participants implicitly associated positive managerial characteristics (i.e., competent, executive, productive) with men. The opposite was true for female participants, who associated women with positive managerial characteristics; however, this effect was much weaker.

Banaji and Greenwald continued to develop understanding of implicit biases including a methodology to reveal individual implicit biases (Banaji, 2001; Banaji and Greenwald, 2013). The Implicit Attitudes Test (IAT) is an empirically sound, popular and well-known tool. Faking bias or non-bias is almost impossible as response time is used (Greenwald et al., 1998; Banaji, 2001). The IAT is available to individuals through implicit.harvard.edu.

A concept related to implicit bias is a *stereotype threat*. This arises when individuals are negatively influenced due to a stereotype within a role or activity. Negative stereotypes affect individuals’ performance when they attempt difficult tasks in the domains in which they are negatively stereotyped (Logel et al., 2012). Stereotype threat can reduce working memory and, because of its relationship with stress, anxiety, and disengagement, can lead to a wide variety of negative outcomes.

### Bias and Career Choice

Both explicit and implicit bias impacts career choice for girls (Lent et al., 1994; Correll, 2001). Biases also impact self-esteem (Greenwald and Banaji, 1995), self-efficacy (Marra et al., 2009; Buse et al., 2013) and stereotype threat (Schmader, 2002). Career choice is impacted by parents, family members and teachers (Lent, 2005).

Media and social media are central to cultural stereotypes, bias, and social norms. Gender bias is pervasive in all forms of media, especially in entertainment. While this phenomenon is well documented, in general, less attention has been paid to women and girls in mathematically demanding fields such as engineering and computing. The depiction of women as professionals, particularly in movies and television is biased because it rarely represents today’s reality. In a study of prime time television shows no women were portrayed as engineers (Geena Davis Institute on Gender in Media, 2014).

Women are more likely to be assigned “supportive” roles in engineering undergraduate teams as opposed to “technical” roles (Laughlin et al., 2007). Women undertaking challenging math problems do equally as well as men but think they have performed worse (Forbes and Schmader, 2010). The academic performance of women students in undergraduate science class is underestimated by their male peers (Grunspan et al., 2016). Faculty members, both men and women, show biases in favor of male students (Moss-Racusin et al., 2012). Students rate male professors higher than female professors. (MacNell et al., 2015), and bias impacts graduation rates and persistence in engineering and computing (Correll, 2001; Buse et al., 2013).

### Evidence-based Interventions

The body of research on the underrepresentation of women in engineering and computing is substantial, yet little research has been done on effective, evidence-based interventions. One area where interventions have been effective has been in recruiting, retaining and graduating women from undergraduate programs. For example, Harvey Mudd College has reached gender parity in graduation rates in their schools of engineering and computing with an intentional, focused effort (Klawe, 2014) and Carnegie Mellon University has increased the number and percent of women with computer science degrees (Frieze et al., 2012). A large Midwest university has successfully changed the admission procedures to increase the number of women recruited to their school of engineering (Holloway et al., 2014). Best practices have been compiled by WEPAN (Women in Engineering ProActive Network, 2016) and NCWIT (National Center for Women and Information Technology, 2016). The ADVANCE program from National Science Foundation has worked to increase the number and percent of women faculty in science and engineering programs by focusing on institutional transformation (National Science Foundation, 2015).

## Discussion: Building Capacity to Accelerate the Research

Gender inequality has been described by researchers as a grand challenge (Joshi et al., 2015). Novel insights, innovation, and a broader community to conduct research are important facets of accelerating the rate of change in women’s representation in engineering and computing. Impacting real world outcomes was a common focus across the discussions among scholars. A number of concrete ideas to build capacity in the research community were put forward including: (1) additional effort to impact the depth and breadth of research studies by using multiple disciplines and multiple methods; (2) leveraging data, merging and mining existing data sources, using data analytics to create knowledge; and (3) providing opportunities for researchers to convene on a regular basis.

## Expanding the Depth and Breadth of Research

An oversight group of researchers and practitioners is recommended to lead the new approach to increasing women’s representation in engineering and computing. The role of the oversight group is to establish priorities, track progress, develop process metrics and outcome measurement while encouraging practitioners and researchers to collaborate. Further, the oversight group could allocate funding and seek new sources of funding. A key responsibility of the group would be to raise consciousness of those in power to understand the bias and barriers limiting women’s achievement in engineering and computing. Since those in power are mostly men, this would include more men in the discourse. The oversight group would develop an effective method to disseminate research into practice in ways that change society, organizations, workplaces, leaders and individual women.

Practice-oriented groups like SIGCS (special interest group on computer science education), WEPAN (Women in Engineering Programs and Advocates Network), SWE (Society of Women Engineers), NCWIT (National Center for Women and Information Technology), Anita Borg Institute, National Academy of Sciences, Engineering and Medicine Committee on Women in Science, Engineering and Medicine, IEEE Women in Engineering and others could be part of the oversight group.

Stakeholders who would benefit from an increased representation of women in the engineering and computing professions are also expected to be part of this group. These stakeholders include corporations, government agencies and other institutions employing those in the engineering and computing professions. Universities that offer engineering and computing degrees and employ faculty in these areas are also important stakeholders.

### Multi-disciplinary

An intentional effort to include multiple disciplines while simultaneously studying multi-levels using multiple methods is essential to accelerate the rate of achievement for women in engineering and computing. The depth and breadth of the research studies will be impacted by using multiple disciplines, multiple methods and simultaneously studying multi-levels.

Currently, the researchers addressing the under-representation are mostly women Ph.D.s in the fields of sociology, psychology, and management. To broaden the impact and the outcomes, the researchers recommend recruiting researchers from additional disciplines. In particular, more economists, engineers, computer scientists, feminist scholars, career scholars, organizational behavior and industrial psychologists are needed in the research studies. Additional researchers who are male would offer a different framework and their participation in this research is encouraged. The role of researchers from practice should be explored as these researchers have access to women who are working in the professions.

### Multiple Methods

The researchers call for studies that utilize multiple types of research methods. An overall framework should be developed, possibly by the oversight group or a specially designated group. From this overall framework multiple theories, methods, and outcome measure could be developed. Types of studies deemed to be important include qualitative, quantitative, ethnographic, meta-analyses, longitudinal, and mixed methods. Employing multiple methods can augment and explain complex or contradictory results, and provide important information on emergent and unexpected themes (Driscoll et al., 2007).

Theoretically framed studies are important so as to build upon prior knowledge and expertise of researchers. More studies should be completed within the workplace so as to understand the complexities of the under-representation of women. Workplace experiences matter as well as context matter in moving the research agenda forward.

Studies of women and men in the workplace are especially needed—studies that include hundreds of firms. Researchers should make contact often and create a longitudinal database. Existing data such as personnel records and performance review can aid understanding of workplace experiences. Social media, blogs, and Facebook and Twitter posts can be used to obtain data. Longitudinal and mixed methods studies are recommended.

It is important that the under-representation of women in engineering and computing be framed and discussed as a societal issue. Too often it has been labeled as a “women’s issue.” The oversight group should emphasize and ensure that the societal impact of women’s equality be emphasized. For example, the problem should be framed in terms of social justice and how the role of women in engineering and computing will impact the wage gap and other social issues related to gendered careers.

Consciousness raising and questioning decisions made by those in power are important but not enough to enable change. The researchers recognize that those in power don’t always understand women’s experience in the workplace.

## Data, Data Mining, and Data Analytics

The under-representation of women in engineering and computing, like any complex problem, can only be solved with a data-driven approach. The new approach to research in this area should use the latest techniques and tools to collect and analyze data. Data, data-based approaches, and data analysis are the foundation of science and technology. The National Academy of Engineering discusses the importance of data or informatics in higher quality and more effective medical care. “As computers have become available for all aspects of human endeavors, there is now a consensus that a systematic approach to health informatics—the acquisition, management, and use of information in health—can greatly enhance the quality and efficiency of medical care and the response to widespread public health emergencies” (National Academy of Engineering, 2016b). The researchers believe this to be true of the work to advance women in the engineering and computing professions. The integration of data and data analysis, especially the role of “big data” and “data analytics” is important according to the researchers to solve the under-representation of women in engineering and computing.

There are numerous national longitudinal surveys of students, doctoral recipients, etc., that are in the area of engineering and computer science (for example, the NSF survey earned doctorates available at^1^). These surveys are under-utilized because (1) researchers do not know they exist, and (2) they are difficult to access. The researchers recommend that an effort be undertaken to locate and assemble all sources of data on students and workers. The effort should include making the existing data easier to find and to use. For example, the Survey of Doctoral Recipients, funded by the National Science Foundation^2^ captures detailed demographic data longitudinally; it has no measures of psychological well-being, no measures that might indicate the impacts of bias, nor any information on future career plans. The researchers recommend adding information related to the micro-processes related to bias, adding a qualitative component and developing a method to randomize participants. This would include identifying people in particular industries, geographic areas, or parents, etc.

*Neurosynth* is a platform for large-scale, automated synthesis of functional magnetic resonance imaging (fMRI) data. It takes thousands of published articles reporting the results of fMRI studies, chews on them for a bit, and then spits out images. It is recommended that a website be developed that compiles the studies on women in engineering and CS, focusing on providing specific information on interventions that enable change.

Data can be compiled from colleges and universities, including, but not limited to admissions offices, registrars, and career services. This data could be available to study differences between men and women, those who persist versus those who leave engineering and CS. Also, alumni offices have data on jobs and organizations of alums.

Corporations collect data on employees, including job progression, training and performance evaluations. Merging the corporate data with other sources could provide a rich source of knowledge on when, how and why women achieve in the workplace.

Specifically, the researchers recommend using and merging existing data sources, leveraging corporate, institutional and governmental databases. A common set of measure and metrics to create knowledge and accelerate change should be created. Novel technologies should be investigated including the use of biometrics and team level data, with a focus on identifying best practices and replication. The scale up from experiments to practice is important along with validity in replicating studies. The use of data mining techniques to identify those times women or girls choose to opt out is important to identify as well as how course content and social media impact achievement. Another recommendation from the researchers is to combine sources of data with micro-factors with larger factors. For example, add psychometric factors to qualitative and quantitative databases. Include demographics, such as number of children that professionals support.

## A Conference of Researchers and Practitioners

The last of the recommendations on changing the approach to research is to provide opportunities for researchers to convene on a regular basis to discuss the status of research, review recent discoveries and identify gaps in knowledge. At the conference, the researchers mentioned over and over again how this was the first time they were able to meet to collaborate with multiple researchers who shared the same research focus. Formal and informal interactions engaged and enlightened the researchers. Collaborations were formed and an agreement to stay in contact were made by many and were included in the comments in the post-conference survey.

The use of social media is recommended as another method to connect researchers. Communities could be established within Facebook or LinkedIn to connect researchers and have an ongoing discussion. Additionally, it could be useful for researchers to intentionally share presentations and status updates. Upcoming conferences and funding opportunities can also be shared.

Connections are necessary with government institutions, with corporations and other stakeholder organizations. Currently, there are limited opportunities for researchers to interact or conduct research within organizations where women are employed as engineers and computing professionals. One exception is women in academic engineering and computing. The ADVANCE program is credited with institutional transformations resulting in increases in the number and percent of women in STEM faculty across the US (National Science Foundation, 2015). Since 2001, the National Science Foundation has awarded more than 300 grants to 200 plus institutions.

Funding should be made available for researchers to attend practitioner conferences and practitioners to attend scholarly conferences. Providing many opportunities for practitioners and researchers to meet and discuss progress will allow interventions to happen organically. The oversight committee can aid in removing barriers to progress.

## Recommendations for Specific Research Studies

This section provides a summary of the specific studies that the researchers recommend to increase women’s representation in engineering and computing. These projects have been grouped into the following categories: (1) Societal and cultural influences on girls and women, (2) Measuring and overcoming implicit bias, (3) Factors influencing career choice in the K-12 environment, (4) Undergraduate and graduate studies, and (5) The workplace.

## Societal and Cultural Influences on Girls and Women

Research is needed to determine interventions are most likely to increase the number of girls selecting engineering and computing professions. Broad-based studies have been suggested by the researchers. These studies must address the intersectionality of gender with other factors, including but not limited to economic status, race and ethnicity, sexual orientation, differently abled and others that may influence career decisions.

The researchers recommended building on current understanding of the emergence of gender roles and gender identity and how these influence career decisions related to engineering and computing. The influence of institutional and organizational factors are important to study, as are non-verbal forms of communication of the gender roles and gender identity. Further, work is recommended on when and how interventions can change current perceptions of gender roles and gender identity related to career choice. For example, studies can follow middle school girls involved in maker camps or coding camps. These types of experiences are proliferating, but longitudinal research studies are necessary to determine the impact on engineering or computing as a career choice.

The impact of government, institutional and organizational policies and the impact of rewards (e.g., to parents, to communities or institutions) are important areas to study. The researchers are interested in understanding the factors that allow some girls to overcome cultural biases and choose engineering and computing. An outcome of this research would include recommendations on how communities can overcome cultural stereotypes related to financial independence where boys are expected to have a career, girls are expected to be mothers.

Researchers recommend that a large-scale effort be coordinated to change the images of engineering and computing. The focus would be to show engineering and computing as inclusive cultures that are professions that change the world. Providing recurring, positive images of engineering and computing as collaborative professions, where women can change the world will change the stereotypes.

The first suggestion to change the stereotypes is to work with marketing and public relations professionals to determine how best to present positive images of a diverse engineering and computer workforce. Positive images of career women portrayed in the media have been credited with an increase in non-traditional career paths. For example, the character of Abby on the television show CSI has created the “Abby Effect” where more girls have chosen forensic science as a career path (Barry, 2012). The public relations work could include additional positive media representations, such as the “Next MacGyver” (The Next MacGyver, 2015). This project is intended to have a female engineer depict the 1980’s character “MacGyver,” a creative inventor and problem solver.

Further, the researchers suggest broad depictions of people working in engineering and computing who are changing the world with their work. For example, Facebook engineers and computing professionals have created a tool where individuals can tell their family and friends they are safe during a disaster (Facebook, 2014). Pre-kindergarten through middle school children can be influenced by various media including games, social media, television, and movies. It is important to ensure that these media sources will provide positive role models for both girls and boys. Because research has shown that parents have the greatest influence on their children’s career choice (Lent, 2005), campaigns targeted at young parents and even at expectant parents would aid in developing a positive and realistic image of engineering and computing as a future career path for any child, whether it be a boy or a girl.

## Measuring and Overcoming Implicit Bias

A limited number of studies have shown that implicit bias exists toward women in science-related careers (Moss-Racusin et al., 2012) and this type of bias can be measured within individuals (Banaji, 2001). Researchers must continue to understand the impact of implicit bias on women in engineering and computing, but a shift is recommended toward understanding how to overcome these types of subtle bias. Specifically, studies are needed on how girls and women recognize and respond to implicit bias in ways that help overcome the bias.

Research projects that are recommended include understanding what can be done to change implicit bias within individuals and how to reduce the impact of other people’s implicit bias on girls and women. Specific research questions related to measuring and overcoming implicit bias include the following. What is the best way for girls and women to respond to implicit bias? Can girls and women intervene to prevent implicit bias from impacting them? Is one intervention effective, or do multiple interventions results in significant change to last a lifetime? Do these interventions need to happen in intervals? Does awareness of one’s biases decrease one’s level of bias? Each of these research questions can only be answered with valid and reliable measurement systems. The researchers recommend leveraging the IAT and continue the development of measurement system to recognize and reduce the impact of implicit bias in individuals and in society.

## Factors Influencing Career Choice in the K-12 Environment

Studies are recommended for understanding which types of programs within the Kindergarten through Grade 12 school environment are most likely to result in more girls choosing engineering and computing. Some research questions are detailed here: Do stand-alone programs work or is it necessary to have multiple experiences throughout the K-12 years? What is the impact of role models, specifically women role models who work in engineering and computing? How best can women in the professions impact students’ career choice? What types of interactions are important to show girls that careers are compatible with women’s lives? How and when should family members have career discussions with children? Are role models who are family members more effective than non-family members? How important is the context of the role model? For example, are family members who are role models more effective than TV characters? When and how are female role models more impactful than male role models? Is the age of role models important to middle school girls?

Clearly identifying pathways to the engineering and computing professions are important research studies. What are the various pathways to success in engineering and computing professions? Are there different rates of success for these different pathways? What is the impact of community colleges and technical vocation programs in high schools? Do these programs lead more girls to choose non-traditional careers in the STEM professions? How do in-school programs targeted at career choice impact the choice of profession especially for girls? How can maker spaces in schools be used to effectively impact career choices related to engineering and computing?

Researchers should work to define age appropriate curriculum in engineering and computing for those in kindergarten through high school. It is recommended that this curriculum include cultural sensitivity, hands-on examples, real-life applications, measurements for learning, teacher and parent guides, and role models in the school. New curriculum should include awareness of how the engineering and computing professions make an impact. An example is the work of engineers and computer scientists in the space program, or in disease detection and prevention. Studies are recommended on how to train teachers to emphasize the connections between important societal problems and the professions of engineering and computing. For example, one of the Engineering Grand Challenges is to provide access to clean water for all people (National Academy of Engineering, 2016a). The impact of these ideas on all children should be studied, including any benefit in attracting a more diverse population to engineering and computing.

Qualitative, longitudinal studies are recommended for children beginning in kindergarten with the purpose of identifying factors that increase and decrease interest in engineering and computing and if it changes over time. It is recommended that researchers study the effect of when students are taking scientific classes and how it impacts career choice. For example, in the U.S., physics is usually taken in the senior year of high school. If moved to a lower grade, would it impact the number of women in engineering?

The integration of engineering and computing into grade schools and the impact on career decisions for both girls and boys is important to study. The researchers recommend longitudinal studies that compare the impact on career choice for integrating engineering and computing concepts into day-to-day coursework versus schools that have separate classes teaching making or coding concepts. Comparing schools which have no programming related to engineering and computing concepts would also provide valuable information on the impact of such a program on career choice for both girls and boys.

Studies are recommended on the work of code.org and Hour of Code and other programs promoting engineering and computer science so as to understand if these interventions are effective in increasing the number of girls who choose engineering or computing careers. The research questions include the following. What types of interventions are most likely to increase interest for girls? How long do the interventions need to be? How can these be integrated into additional schools and communities? What are the characteristics of those interventions that result in more girls choosing engineering and CS careers?

A final recommendation from the researchers is based on feminist studies which hypothesize that there is really no such thing as completely objective or unbiased work. But currently, science and math are taught in ways that are objective and fact-based. The subjectivity of concepts is minimized. The researchers recommend creating a research agenda that explores how objective and fact-based curriculum impacts the success of students, particularly girls and other under-represented minorities. The study should explore methodologies where scientific work is viewed in a more subjective manner. This could be done by engaging students in a discussion of the subjectivity within the sciences and math fields. For higher level courses in computer science and engineering, research is recommended where students are taught to think more about the objectivity and subjectivity in these fields. Will this type of study open minds to the different kinds of work that can be accomplished? And will it allow students to have more ownership over their own biases as part of the way of doing the work?

## Undergraduate and Graduate Programs

While there is much known about the complexity of problems facing women in undergraduate and graduate programs, research offers few practical strategies to increase the recruitment, retention, and graduation of women in engineering and computing. Beginning with the recruitment process, the impact of recruitment mailings, advertisements, emails, etc., should be assessed. Campus tours, admissions processes and other aspects of recruiting should be studied to understand biases. Instituting common metrics across all programs can aid in developing an understanding of differences. Further studies should investigate how the use of outcome measurements could be used to change institutional policies.

Longitudinal studies are recommended for all students, both women and men, in undergraduate and graduate programs to understand retention leading to graduation. At this time, it is not well understood when and if specific classes, projects, team projects, student groups, faculty interactions, etc., impact persistence. The studies should focus on those specific programs, experiences, networks, methodologies, and other opportunities that influence women’s persistence in academia. Additionally, research should address understanding the factors within undergraduate and graduate programs that are linked to continued achievement in the engineering and computing workplace. Understanding how these experiences and influences are the same or different from their male classmates is important to influence the under-representation of women.

It has been shown that having more women as professors helps women persist (Klawe, 2014) but we need to better understand how all professors can differentially develop skills to enable persistence for all students. Supporting relationships have been shown to allow all undergraduates to persist, understanding how these relationships could be leveraged to achieve better outcomes for women is an important research opportunity. These relationships should include professional women or other role models, mentors and sponsors.

Self-efficacy (Betz and Hackett, 1986) and professional role confidence (Cech et al., 2011) have been shown to impact career choice and persistence in undergraduate programs. Researchers should focus on how women develop these individual characteristics during the undergraduate experience. An important aspect of this research is to understand the impact of other factors, including race, ethnicity, sexual orientation, income, etc., on these individual characteristics.

A suggestion was made to begin a large-scale randomized controlled study across universities, for example in all of the Big 10 schools and in selected smaller schools. Universities would be willing to participate as this type of study will improve the student’s experience which may eventually increase alumni donations. This large-scale study would focus on persistence across different educational experiences—large universities, private, and community colleges. The study can incorporate geographical differences, campus differences (for example those that have an athletic focus vs. those that do not), tuition aid, traditional vs. non-traditional students, etc. Ideas for further development include rewarding women and minorities for taking engineering and computing programs. The reward could be an internship, promise of a job post-graduation, money, grants or other tangibles.

## The Workplace

Because researchers have limited access to engineering and computing workplaces, studies on professionals working in engineering and computing careers are limited. The one exception is in the academic workplace where the ADVANCE program (National Science Foundation, 2015) has successfully increased the participation and advancement of women in academic science and engineering careers. Future research studies are suggested that focus on women in professional engineering and computing careers so as to understand what interventions are needed to recruit, retain and advance women in these professions. Having an oversight group of practitioners and scholars would facilitate the research within workplaces.

Prior work shows why women leave the engineering and computing professions (Singh et al., 2013; Fouad et al., 2015) and identifies factors related to women persisting in these careers (Buse et al., 2013; Buse and Bilimoria, 2014). Moving forward, the researchers recommend that studies focus on how to make workplaces free from bias, stereotypes, and micro-aggressions. Men and women should be trained to understand how bias, barriers, and micro-aggressions manifest themselves within workplaces. Research is needed on how to minimize and/or eliminate the bias, barriers, and micro-aggressions. For example, some companies are using “blind” recruitment processes, where names are taken off applications and resumes. Studies are needed to understand how longer term biases can be overcome. An understanding of backlash is needed where the backlash is an adverse response to bias training and/or promotional opportunities provided to women.

Sponsors and mentors have been shown to be effective in the retention and advancement of women in leadership (Singh et al., 2006). Researchers should understand the optimal types of sponsors and mentors for women in engineering and computing. Studies show that women are more likely to persist in engineering when they are engaged with their work and find meaningfulness with their career (Buse and Bilimoria, 2014). These factors can be used to attract and retain women to engineering and computing. Specific studies are suggested to understand if workforce diversity is increased when workplaces recruit based on a “change the world” strategy and if the work actually impacts the world positively. There is an opportunity by workplaces to ensure that the job itself is linked to a higher purpose, and researchers can aid practitioners in crafting a higher purpose within organizations.

Researchers should examine women’s work and men’s work within engineering and computing to determine how work is distributed and enacted so as to overcome recent studies that show women are more likely to work in supporting and less challenging roles and do the office housework (Williams and Dempsey, 2014). Some disciplines like biomedical engineering attract and retain more women than other disciplines like electrical engineering. Researchers are encouraged to explore the nuances involved in the professions to better understand these phenomena. Different disciplines within engineering and computing need to be studied and compared. Within engineering and computing there is much diversity, in the type of work, in the workplace itself (e.g., oil rigs vs. offices) in the specific industries (e.g., academia vs. industry vs. government), in organization size (e.g., Google vs. start-up) etc. Researchers are encouraged to understand these sub-fields and gender distribution within the subfields. The exploration of these differences is recommended so as to discover if there is a socio-technical divide. Work is recommended to show society how and when engineering and computing professions serve the higher good. The identification of organizations, industries, and sub-fields that serve the public can help engage more individuals within the professions.

The researchers suggest studies on the language of engineering and computing as some disciplines have a clear male-female dynamic within the language. For example, in the chemical industry hose fittings are described as male or female. The impact of language within professions and industries and how it impacts the retention and advancement of women in various engineering disciplines, and various computing disciplines should be studied.

Comparative studies are recommended between other professions such as medicine and law where women have greater representations. More studies are needed to understand the differences that men and women experience within the engineering and computing workplace. These studies should focus on the specific changes that organizations can implement to integrate women into the existing workplace while implementing longer term strategies to obtain gender equity.

Researchers should determine the types and frequency of educational initiatives for organizational leaders on bias, barriers, and stereotypes. Organizational leaders should work with experts in implementing structural changes to increase women’s representation in engineering and computing. Institutional and governmental policies should be identified that can be used to impact women’s achievement in the engineering and computing professions.

A comparative study is recommended where researchers identify two groups of organizations: ones that have had success in retaining and advancing women in engineering and computing and other organizations that are unable to retain women. Identify the type of work, the organizational policies and practices and other factors that differentiate the two. This may include the type of professional and leadership development for the managers, team structures, and work opportunities for the women, and differences between those in powerful positions.

Researchers should determine how work location, work hours, work schedules, and virtual work impacts the achievement of professional women. Longitudinal studies are necessary, especially those that include the work and home interface (e.g., the role of the husband in doing housework, childcare, etc.).

Work is needed to bring together firms employing engineers with undergrad programs to identify what is needed in the curriculum to achieve as an engineer or in computing. Time and effort should be expended to review and update courses and curriculum to reflect today’s work environment. This can only be done with teams of men and women from practice working with those in academia.

Organizations can create survival guides for women in engineering and computing. Another suggestion from the researchers is to create a ranking system for good places for women engineers and computer professions. This would be similar to the US News and World Report rankings of the best colleges. And the last recommendation is to understand how counter-spaces or work groups of the same races or ethnicity impact women’s achievement in professional engineering and computing.

## Limitations

While the conference organizers attempted to include a range of disciplines studying the under-representation of women in engineering and computing, it is clear that several fields were missed. Not included were any feminist scholars or researchers from economics.

Another limitation of this report is that it only includes “what to do” not the “how to do” needed to enact the research agenda. The work of the conference organizers and the researchers moving forward is to find the opportunities to establish how to implement the many suggestions identified and documented here.

## Conclusion

Researchers had a unique opportunity to gather and discuss their efforts to understand the factors impacting the under-representation of women in the engineering and computing professions. The researchers agreed that a new collaborative research approach is necessary to accelerate the rate of change in women’s representation in these important professions. Recommendations for moving forward include the creation of an oversight committee comprised of researchers, practitioners and other stakeholders, developing a network including additional disciplines applying new technologies and multiple methods to leverage data and data analytics, and to provide continuing opportunities for collaboration among multi-sector and multi-discipline stakeholders to implement effective interventions.

Additionally, the researchers suggested a future research agenda focusing on socio-cultural influences, measurement and intervention strategies related to girls and women at all stages of the career choice process. The under-representation of women in engineering and computing should be viewed as a complex problem that can be overcome with a collaborative strategy, appropriate resources including funding and stakeholders working together to implement effective solutions.

## Author Contributions

KBu is main author. CH and KBe wrote several sections.

## Conflict of Interest Statement

The authors declare that the research was conducted in the absence of any commercial or financial relationships that could be construed as a potential conflict of interest.

## Footnotes

1 https://www.nsf.gov/statistics/2017/nsf17306/survey.cfm

2 https://www.nsf.gov/statistics/2017/nsf17306/survey.cfm
